# Supplementary material for: Incidence, healthcare-seeking behavior and barriers associated with seeking care for severe childhood pneumonia in rural Bangladesh: A prospective study
Source: PLOS Glob Public Health. 2025 Mar 10;5(3):e0004105. doi: 10.1371/journal.pgph.0004105 (PMC11893127; doi:10.1371/journal.pgph.0004105)
Supplement: Attachment — Submitted filename: Response to Reviewers.docx [file pgph.0004105.s003.docx]

Reviewer #1: Overall, it is a good paper that is showing reduced incidence in pneumonia mortality as well as improved care seeking behaviour in a developing country. However, the authors need to address the following:
1. Line 145, the should remove word "therefore" on the opening of the sentence

***Response:*** *Thanks a lot for spending your valuable time for reviewing the manuscript. Removed the word, therefore.*

2. Lines 203 -207, the authors should clearly state what they were studying "families or children with pneumonia?", the authors should drop the sentence "chest indrawing was the leading complaint, it will confuse readers"

***Response:*** *Thank you very much for your valuable comment and suggestion. We excluded families to avoid confusion. Also, we dropped the sentence "chest indrawing was the leading complaint.*

3. Lines 225-226, the percentage of males and females should add to 100% unless otherwise

***Response:*** *Thanks for your concern. It was the distribution of severe pneumonia among males vs females based on their different age categories. If we sum the column percentage in each column it will add up to 100%. To avoid confusion, we mentioned the age group in the text with the % distribution of severe pneumonia between males and females.*

4. table 3, make the table tidy, remove word indicator and make a good title instead of inserting too much words in the table. there are some confusing figures in the last column of table 3.

***Response:*** *We revised Table 3 according to your suggestion. We cross-checked the numbers and percentages in Table 3 and found them fine.*

5. the authors should discuss the results "result by result" for this to be very clear. it will be interesting to discuss why is the incidence declining, there should be articles especially on pneumococcal vaccine reducing incidence. on the care seeking behaviour, discuss it properly so that they inform the readers why it has reduced.

***Response:*** *Thank you so much for your valuable suggestions. We have revised the discussion section accordingly.*
