# Supplementary material for: Incidence, healthcare-seeking behavior and barriers associated with seeking care for severe childhood pneumonia in rural Bangladesh: A prospective study
Source: PLOS Glob Public Health. 2025 Mar 10;5(3):e0004105. doi: 10.1371/journal.pgph.0004105 (PMC11893127; doi:10.1371/journal.pgph.0004105)
Supplement: Attachment — Submitted filename: final comments.docx [file pgph.0004105.s002.docx]

Thank you for your trust in me to review this paper.

The authors have addressed all the comments and the discussion section has now greatly improved.

very minor issues: line 217 remove word "highest" and line 392-3, this will be more valid if National vaccination coverages for pneumonia related vaccines are presented just in a sentence" or an average over the past 3 years.
