# Supplementary material for: Incidence, healthcare-seeking behavior and barriers associated with seeking care for severe childhood pneumonia in rural Bangladesh: A prospective study
Source: PLOS Glob Public Health. 2025 Mar 10;5(3):e0004105. doi: 10.1371/journal.pgph.0004105 (PMC11893127; doi:10.1371/journal.pgph.0004105)
Supplement: Attachment — Submitted filename: Response_to_Reviewers_auresp_2.docx [file pgph.0004105.s004.docx]

Thank you for your trust in me to review this paper.

The authors have addressed all the comments and the discussion section has now greatly improved.

very minor issues: line 217 remove word "highest" and line 392-3, this will be more valid if National vaccination coverages for pneumonia related vaccines are presented just in a sentence" or an average over the past 3 years.

***Response:*** *Thanks for your valuable time for reviewing the manuscript. We corrected the minor issues in the revised version.*

Reviewers' comments:

Reviewer #1: (No Response)

Reviewer #2: The study focuses on incidence of severe pneumonia and barriers to access to care for children with severe pneumonia. The survey was conducted at household level.

The authors should consider the following comments to make the manuscript clearer.
The methods section needs to be improved upon to make it easy for the reader to understand and objectively assess the rigor of the study and validity of the results. The authors need to provide clarity on the following:
Study design: The study was nested in a trial, but there is no information about the trial except the link to the site where the trial was registered. A summary with relevant information about the trial and how the current study was nested in there should be provided.

***Responses:*** *Thanks for your valuable comment. We have added the objective of the main trail and also added a link of published article from this trial. After the initiation of the cluster-randomized control trial (CRCT), the study investigators were intended to know the incidence of severe pneumonia. Therefore, they conducted this descriptive epidemiological study but it wasn’t influenced the CRCT. The revision is below—*

*“This trial aimed to define the clinical and cost-effectiveness of a Day Care Approach alternative to Usual Care (comparison group) within the Bangladesh health system to manage severe childhood pneumonia. The results of this RCT were published eClinicalMedicine journal (*[*https://www.thelancet.com/action/showPdf?pii=S2589-5370%2823%2900200-6*](https://www.thelancet.com/action/showPdf?pii=S2589-5370%2823%2900200-6)*)”*

The cases are described as children with severe pneumonia and controls those with non-severe pneumonia. This implies a case-control study design. The authors need to explain how the case-control study was nested in the trial.

***Responses:*** *It wasn’t a case-control study rather it’s descriptive epidemiological study and data were collected prospectively from both control and interventions clusters with a view to find the incidence of severe pneumonia with a duration of one-year period.*

In the introduction, the authors indicated that they wanted to collect data on children with severe pneumonia who do not present to the health care settings like health centres and hospitals but remain in the community, to understand the barriers to health seeking. However, in the methods section, it is not clear how these children were identified.

***Responses:*** *The children were identified by the door to door households visit having at least one under-five children in the households. Added in the manuscript. (page 6-7; lines 163-174)*

The sampling strategy was stated to be multi-stage but this requires more details. In this survey, how did the team do the sampling from the districts to the households? And how does this relate to the main trial and the case-control study design of the current study?

***Responses:*** *We have added the sampling strategies in details. This study was separate from the main trial. The main objective of this descriptive study was to find out the incidence of severe pneumonia. (page 6-7; lines 163-174)*

There is data that was obtained from the health facilities, other sources of care and from households. The authors need to provide clarity on the different data sources and
Clarity on what part of the study was prospective and retrospective will help the reader to gain better understanding of the study results.

***Responses:*** *To enhance the number of respondents and capture maximum information as much as possible, we collected data from various sources. Few data including the demographic characteristics and health-care seeking behavior were collected from household but the clinical characteristics data of the same children were collected from the physicians.*

The primary aim of the study was to obtain data on the incidence of severe pneumonia. Whereas there is some information on how the data on incidence was obtained (although this requires more information), there is no description on how the data on barriers to care-seeking for children with severe pneumonia was obtained.

***Responses:*** *This study only identified the new episodes in last one years in the households using structured questionnaire. (page 7; lines 178-191)*

There is some information missing on sample size calculation. The formula used is not stated which makes it difficult to assess if the right formula for incidence was used.

**Responses:** We revised the sample size calculation. Below is the revision—

*The study captured significant variability because information was collected from numerous households both intervention and control clusters (unions) across all study sites (sub-districts). The sample size was calculated using the formula,* $n=\frac{Z_{\alpha}^{2} P(1-P)}{d^{2}}$*, where p is the incidence of severe pneumonia in under-five children (2.02%), Z_α_ is 1.96 at a 5% level of significance, and d is the desired precision (0.5%). Using these parameters, the minimum sample size was determined to be 3042. Considering a design effect of 3 and a 10% non-response rate, the total sample size required was 10040 children under 5 years old. The study also tracked severe pneumonia episodes over a 12-month period. It was assumed that during this period, about 80% of severe pneumonia cases would be captured from HFWCs, sub-district hospitals, and district hospitals, as distance is a significant factor in seeking health care. The survey estimated the number of new cases by collecting information using both prospective and retrospective approaches, with the population size serving as the denominator in the past year.*

The statistical analysis needs improvement. Which data were analysed for the different objectives? What were the outcomes of interest? Which method analysis was used?

***Responses:*** *Revised and added more information. (page 7-8; lines: 195-213)*

Results
The results present information from households about children in general and those with severe pneumonia. There are also results about children seen by the physician. It is difficult to objectively assess these results because the methods used to collect the data that informed the results are not clear.

***Responses:*** *Yes, you are right. Few data including the demographic characteristics and health-care seeking behavior were collected from household but the clinical characteristics data of the same children were collected from the physicians.*

Results of incidence of severe pneumonia in the control group are presented. However, in the section on study design, the authors indicated that the controls were non-severe pneumonia cases. It is therefore confusing the have severe pneumonia incidence in the control group.

***Responses:*** *We have corrected the study design section. In this study, we collected data both from control and intervention clusters to find out the incidence of severe pneumonia and this study is based on this objective in addition to explore the health care-seeking behaviour.*

This study seems to have been primarily about incidence of severe pneumonia and barriers to healthcare seeking. The clinical characteristics of the 154 children with severe pneumonia are presented in the tables, disaggregated between males and females. It is not clear how this information relates to the core problem under study (barriers to care-seeking). It is recommended that the data is presented in this context. For example, among those children with cases and controls, how many of them did not seek care, how many delayed to seek care, what were the barriers to seeking care?

***Responses:*** *We have rearranged the results section according to the study objectives.*

Results on barriers to care-seeking are presented but the methods that were used to collect these data were not described.

***Responses:*** *We have added in the method section. (Page 7; lines 178-191)*

In the discussion, the authors indicated that the caregivers had no difficulty accessing care, yet at the same time, they show that there were financial barriers. These issues are contradictory. Probably operational definition of access to care and barriers to care would help.

***Responses:*** *We have revised the discussion section.*

Clarity on the methods and results is required to make an objective assessment of the discussion.

***Responses:*** *We have rearranged the methods and results section according to the objectives.*

Reviewer #3: The study title explores an important question regarding health seeking behavior and its barriers for severe childhood pneumonia in Bangladesh. The manuscript describes some components but is difficult to follow.

Introduction - has irrelevant text (lines 63-78;82-85;95-108), cites literature10 years or older and does not focus on what is the current status in Bangladesh

***Responses:*** *We have updated the reference and revised the introduction.*

Most importantly, how do the authors justify care seeking in a study that is nested in a cluster RCT where participants are expected to report to study clinics with severe pneumonia?

***Responses:*** *This study was separate from the main trial. The main objective of this descriptive study was to find out the incidence of severe pneumonia. From the descriptive study the participant may seek care to the main trial but we don’t have this information how many children included in the main trial who actually also participated in this prospective epidemiological study.*

Methods:

How was data on incidence collected is also not clear with unclear information on time of follow up. Especially with regard to additional data on pneumonia reporting to other facilities
Under the heading 'study setting' recruitment procedures are described rather than providing a description of population characteristics and geography of the chosen districts

***Responses:*** *We didn’t collect the follow-up data from the participant. From the households we collected data from their care-seeking.* *(Page 7; lines 178-191)*

How was information on barriers to care seeking behavior (third objective) collected is not clear

***Responses:*** *Data were collected from household surveys with structured questionnaire. (Page 7; lines 178-191)*

Sample size calculation is not clear.

**Responses:** We revised the sample size calculation. Below is the revision—

*The study captured significant variability because information was collected from numerous households both intervention and control clusters (unions) across all study sites (sub-districts). The sample size was calculated using the formula,* $n=\frac{Z_{\alpha}^{2} P(1-P)}{d^{2}}$*, where p is the incidence of severe pneumonia in under-five children (2.02%), Z_α_ is 1.96 at a 5% level of significance, and d is the desired precision (0.5%). Using these parameters, the minimum sample size was determined to be 3042. Considering a design effect of 3 and a 10% non-response rate, the total sample size required was 10040 children under 5 years old. The study also tracked severe pneumonia episodes over a 12-month period. It was assumed that during this period, about 80% of severe pneumonia cases would be captured from HFWCs, sub-district hospitals, and district hospitals, as distance is a significant factor in seeking health care. The survey estimated the number of new cases by collecting information using both prospective and retrospective approaches, with the population size serving as the denominator in the past year.*

Line 193 - presenting 95% CI as inferential measure is unclear

***Responses:*** *We excluded the 95% CI measure since it is a descriptive study and we presented only the descriptive measure.*

Results: There is a lot of irrelevant data presented in tables and text
Overall severe pneumonia incidence 0.013 (95% CI:0.25-0.28) mentioned here and in the abstract and elsewhere is probably a typo and needs correction.

***Responses:*** *You are right, it was typo. We apologies for this unintentional error. We have corrected in the revised version.*

What is the rationale for presenting severe pneumonia incidence in intervention and control clusters and age categories (Tables 2 and 3)?

***Responses:*** *The incidence was presented in overall and in control and intervention clusters to see the overall incidence as well as incidence in the control and intervention clusters. We excluded the age category-wise distribution of incidence. However, we distributed the demographic characteristics among the severe pneumonia children.*

Table 3, 4 and 6 and description in lines 241-246; 253-256 do not match

***Responses:*** *We apologies for this unintentional error. We have rearranged the tables and results sections.*

Lines 265-280 provide irrelevant text and data that are not part of the objectives

***Responses:*** *We excluded the irrelevant sentences.*

Discussion - very lengthy and difficult to follow.

***Responses:*** *We have revised the discussion section.*

Lines 304-312 describes delayed/incomplete immunization as a determinant for severe pneumonia but fails to explore why this is so from the parent's perspective, which is an objective that authors are trying to study

***Responses:*** *We have added expanded more in the revised version.*

Lines 337-346 also does not address the objectives of the manuscript but rather deviates from it.

***Responses:*** *We have rearranged and revised the discussion section.*
