# Supplementary material for: Incidence, healthcare-seeking behavior and barriers associated with seeking care for severe childhood pneumonia in rural Bangladesh: A prospective study
Source: PLOS Glob Public Health. 2025 Mar 10;5(3):e0004105. doi: 10.1371/journal.pgph.0004105 (PMC11893127; doi:10.1371/journal.pgph.0004105)
Supplement: Attachment — Submitted filename: Response_to_Reviewers_auresp_3.docx [file pgph.0004105.s005.docx]

Reviewer #3: Thank you accepting the suggestions and comments and attempting to address them. Despite this, there are still some significant lacunae that are outlined below
1. The incidence which was calculated and presented correctly (except for the 95% limits) in the first version has now been changed with no reason given why this was done. The current figure although reported as incidence seems more like the prevalence. Similarly the sample size calculations have used prevalence. This needs to be addressed.

***Response:*** *Thank you very much for your time and for your critical yet valuable review. We truly appreciate it. As this was a descriptive study, we felt that presenting inferential statistics, such as 95% confidence intervals, was not necessary, so we excluded them from the manuscript. Since we prospectively collected data and diagnosed severe pneumonia cases based on signs and symptoms, these were classified as new cases, and by definition, represent incidence (new cases divided by the total population at risk). This is why we referred to it as incidence. Also, to make the incidence rate easier for readers to understand, we multiply it by 100 to express it as a percentage. The sample size calculation for both incidence and prevalence is the same, and we conducted the sample size calculation based on incidence.*

2. The three main objectives of this study as reported in the manuscript are
- incidence of severe pneumonia
- care-seeking behavior
- barriers to care seeking
The results if presented according to these objectives makes it easier to understand and read. Presenting results by intervention and control groups makes it very confusing. Likewise, it is difficult to comprehend the distribution of clinical signs based on gender. The table with antibiotics used for treatment of pneumonia is also not part of the study objective and not clear why this has been described.

***Response:*** *Thank you once again for your constructive comments. Following your suggestion, we have reorganized the results to align with the study objectives. We fully agree with your point and have excluded the control and intervention groups, presenting the overall results instead. We retained the section on clinical signs before introducing the incidence, as readers may be interested in the percentage distribution of clinical signs. Although we kept the sex distribution due to the commonly observed differences in care-seeking behavior by sex, we focused on presenting the overall results. We also agree with your feedback regarding antibiotics and drug use, which are not part of our study objectives, and have therefore excluded this section from the results.*

3. Discussion - this section has been improved but only partially. The second and third paragraph (Lines 312 - 346) has text and findings of studies that are not part of the this study's objectives. While the authors have found some relevant findings related to care seeking and its barriers, discussion regarding the significance of these findings, analyses of why and what could be the ways to overcome them with recommendations is missing.

***Response:*** *Lines 312-346 are based on incidence of severe pneumonia that aligns with one of the study’s objectives, so we will retain this section. Following your suggestion, we have added an explanation of we included the explanation of why and what in the discussion section. Additionally, we have included recommendations in the discussion section to enhance clarity and relevance.*
